# SARS-CoV-2 Proteins Interact with Alpha Synuclein and Induce Lewy Body-like Pathology In Vitro

**DOI:** 10.3390/ijms23063394

**Published:** 2022-03-21

**Authors:** Zhengcun Wu, Xiuao Zhang, Zhangqiong Huang, Kaili Ma

**Affiliations:** 1 Institute of Medical Biology, Chinese Academy of Medical Sciences and Peking Union Medical College, Kunming 650118, China; wzc1121@imbcams.com.cn (Z.W.); xiuaonb@student.pumc.edu.cn (X.Z.); hzhq@imbcams.com.cn (Z.H.); 2 Medical Primate Research Center & Neuroscience Center, Chinese Academy of Medical Sciences and Peking Union Medical College, Beijing 100005, China

**Keywords:** Parkinson’s disease, SARS-CoV-2, alpha-synuclein, spike protein, nucleocapsid protein

## Abstract

Growing cases of patients reported have shown a potential relationship between (severe acute respiratory syndrome coronavirus 2) SARS-CoV-2 infection and Parkinson’s disease (PD). However, it is unclear whether there is a molecular link between these two diseases. Alpha-synuclein (α-Syn), an aggregation-prone protein, is considered a crucial factor in PD pathology. In this study, bioinformatics analysis confirmed favorable binding affinity between α-Syn and SARS-CoV-2 spike (S) protein and nucleocapsid (N) protein, and direct interactions were further verified in HEK293 cells. The expression of α-Syn was upregulated and its aggregation was accelerated by S protein and N protein. It was noticed that SARS-CoV-2 proteins caused Lewy-like pathology in the presence of α-Syn overexpression. By confirming that SARS-CoV-2 proteins directly interact with α-Syn, our study offered new insights into the mechanism underlying the development of PD on the background of COVID-19.

## 1. Introduction

The severe acute respiratory syndrome coronavirus 2 (SARS-CoV-2) outbreaks known as the 2019 novel coronavirus disease (COVID-19) pandemic has caused high levels of concern and economic crisis around the world [1]. Previous studies have supported a link between SARS-CoV-2 infection and Parkinson’s diseases (PD) [2,3,4,5]. More recently, several cases of patients with COVID-19 who developed parkinsonism and responded to levodopa have been reported. However, none of those patients had any history suggestive of parkinsonism or taking medications that could lead to secondary PD before developing a COVID-19 infection [6,7,8,9,10]. One of these cases was a 45-year-old Israeli patient who developed PD soon after hospitalization due to SARS-CoV-2 infection [10]. There is growing evidence in PD patients suffering from COVID-19 that COVID-19 could worsen PD [4], suggesting that COVID-19 might be associated with an elevated long-term risk of PD.

PD is the second most common neurodegenerative disease; it accounts for 5/100,000 to more than 35/100,000 new cases each year [11]. It is characterized by a progressive loss of dopaminergic neurons in the substantia nigra pars compacta and the presence of Lewy bodies (LBs) in numerous brain regions [12]. Similar to some other viral infections, one of the possible outcomes of COVID-19 might include pathological changes in the brain, which might accelerate neurodegeneration due to the increase protein aggregation in the brain. A protein–protein docking research confirmed the interaction between SARS-CoV-2 spike (S) protein and amyloidogenic proteins [13]. Therefore, exploring the interaction of viruses or viral particles with the brain proteins might offer new insights into molecular links between COVID-19 and PD.

SARS-CoV-2 contains four structural proteins. The nucleocapsid (N) protein and spike (S) protein are most abundant in SARS-CoV-2 particle [14]. S protein plays a key role in the receptor recognition and cell membrane fusion process. It is composed of two subunits, S1 and S2. The S1 subunit contains a receptor-binding domain (RBD) that recognizes and binds to the host receptor angiotensin-converting enzyme 2 (ACE2). Owing to its indispensable function, it represents one of the most important targets for COVID-19 vaccine and therapeutic research [15,16]. The N protein is an abundant RNA-binding protein critical for viral genome packaging and plays a critical role in the regulation of cell signaling pathways [17]. The N protein of SARS-CoV-2 can be divided into five domains: a predicted intrinsically disordered N-terminal domain (NTD), an RNA-binding domain, a predicted disordered central linker, a dimerization domain, and a predicted disordered C-terminal domain (CTD). The N protein is also considered a target for vaccine development because in the SARS family of viruses, the N protein gene is more conserved and stable than the S protein gene [18].

Alpha synuclein (α-Syn) coded by *SNCA* genes, is a highly conserved 140-amino-acid protein. It is mainly located at presynaptic terminals and is expressed uniquely in neurons of the central and peripheral nervous systems (CNS and PNS) [19]. It is considered a key protein in PD pathogenesis, given that abnormal proteinaceous aggregates of α-Syn are the main component of LBs—the neuropathological hallmarks of PD [20]. We speculated that a molecular interaction between virus proteins and α-Syn might illustrate the link between SARS-CoV-2 and PD. Therefore, in this study, we investigated the direct interaction effects of SARS-CoV-2 proteins with α-Syn by means of bioinformatics analysis and cells level. Our research may provide new insights into selective vulnerability of COVID-19 patients to PD.

## 2. Results

### 2.1. Protein–Protein Interaction between SARS-CoV-2 Proteins and α-Syn

**Figure 1:**
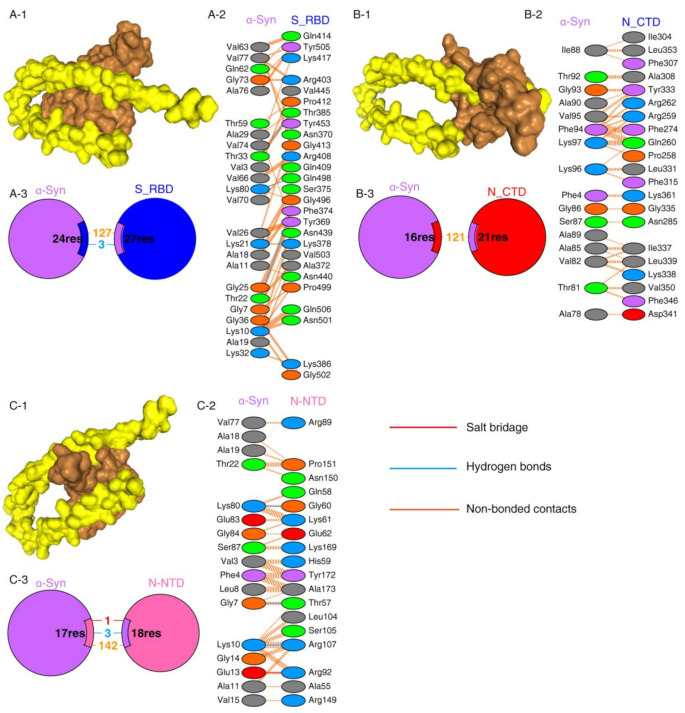
SARS-CoV-2 proteins’ interaction with α-Syn by protein–protein docking. (**A**) The protein–protein docking result of S_RBD (PDB ID: 1XQ8) and α-Syn. (**A-1**) Docking model showing the interaction of S_RBD (brown) and α-Syn (yellow). (**A-2**) Detail of the interacting residue of S_RBD and α-Syn. (**A-3**) Pie chart shows the key interaction of residues between S_RBD and α-Syn. (**B**) The protein–protein docking result of N_CTD (PDB ID: 6WJI) and α-Syn. (**B-1**) Surface diagram of N_CTD (brow) and α-Syn (yellow) complex model. (**B-2**) Detail of an interacting residue of N_CTD and α-Syn. (**B-3**) Pie chart shows the key interaction of residues between N_CTD and α-Syn. (**C**) The protein–protein docking result of N_NTD (PDB ID: 6VYO) and α-Syn. (**C-1**) Docking model of interaction of N_NTD and α-Syn. (**C-2**) Detail of an interacting residue of N_NTD and α-Syn. (**C-3**) Pie chart shows the key interaction of residues between N_NTD and α-Syn. Key interactions between residues are presented as dotted lines. The key interactions are color coded as follows: salt bridge (red), disulfide bonds (yellow), hydrogen bonds (blue), and non-bonded contacts (orange). The number of lines indicates the potential number of bonds. For non-bonded contacts, the width of the striped line indicates the number of potential contacts.

Protein–protein interactions are a crucial prerequisite for many biological interactions involved in cellular signaling, immunity, and cellular transport [21]. Potential interactions between the SARS-CoV-2 proteins and α-Syn were examined by the HDOCK server. Model 1 with the highest docking energy score and the lowest ligand RMSD was selected. The docking results are shown in Figure 1 and Table 1. As shown by the docking scores in Table 1, the increasing affinity of proteins toward α-Syn was as follows: N-CTD > S1-RBD > N-NTD. PDBSum was used to determine the interacting residues of the protein complexes. The interacting surfaces and the binding residues are shown in Figure 1. The docking results showed that the interaction of α-Syn and S1 (docking score: −243.43) was mediated by three hydrogen bonds through Thr33, Lys80, and Thr22 residues with Thr385, Gln498, and Phe 374 of S1 protein (Figure 1A) and 127 of non-bonded contacts. The N-CTD–α-Syn complex (docking score: −252.56) showed its interactions with contribution of 121 non-bonded contacts (Figure 1B). α-Syn formed three hydrogen bonds and one salt bridge with N-NTD (docking score: −209.03). The hydrogen bonds are formed between Lys80, Gly7, and Lys10 of α-Syn to Gly60, Thr57, and Arg107 of N-NTD. The only salt bridge formed between Glu13 of α-Syn and Arg 107 of N-NTD protein (Figure 1C), suggesting a more favorable interaction of α-Syn with N terminal than C terminal of N protein.

The binding affinities of docking structures represented by dissociation constant (Kd) were obtained by PPA-Pred2 (Table 1). The lower the Kd, the higher the affinity. As shown in Table 1, the binding affinities of α-Syn complexes showed that α-Syn–S1 had a strong binding affinity (1.46 × 10^−08^ M) among other complexes, followed by α-Syn–N_NTD (2.17 × 10^−07^ M) and α-Syn–N_CTD (4.72 × 10^−07^ M).

### 2.2. α-Syn Directly Interacts with SARS-CoV-2 Proteins in HEK293 Cells

**Figure 2:**
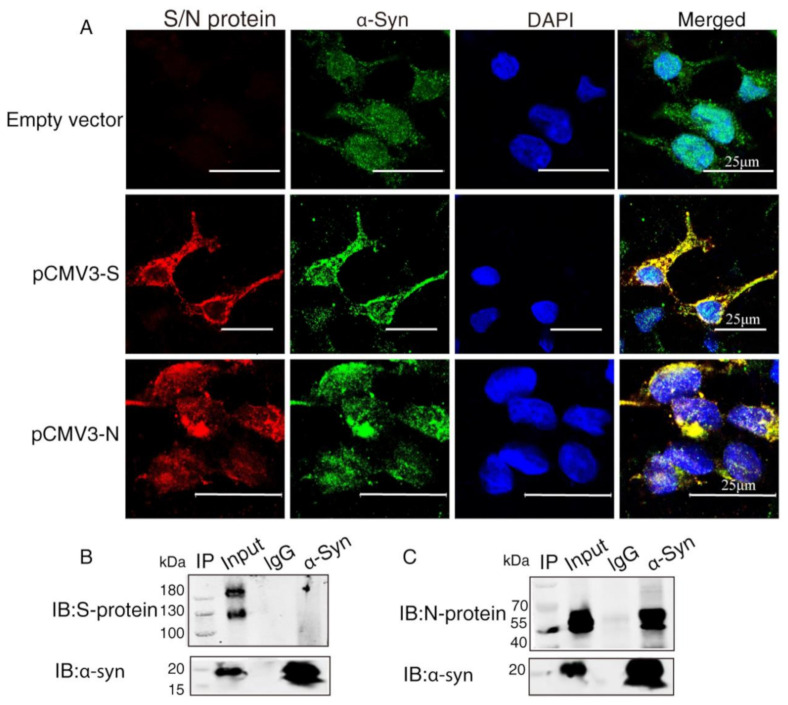
α-Syn directly interacts with SARS-CoV-2 proteins in HEK293 cells. (**A**) The distribution and location of SARS-CoV-2 proteins and α-Syn in HEK293 cells, as analyzed by confocal microscopy. DAPI was used to stain nuclei. (**B**) Interaction between endogenous SARS-CoV-2 S protein and α-Syn in HEK293 cells. Cell lysates of HEK293 cells co-transfected with S protein and α-Syn were prepared and used for Co-IP. The coimmunoprecipitates were analyzed by Western blotting with anti-α-Syn. (**C**) Interaction between endogenous SARS-CoV-2 N protein and α-Syn in HEK293 cells. Cell lysates of HEK293 cells co-transfected with N protein and α-Syn were prepared and used for Co-IP. The coimmunoprecipitates were analyzed by Western blotting with anti-α-Syn.

To investigate the interaction between α-Syn and SARS-CoV-2 proteins, we co-transfected the recombinant plasmids (pCMV3-S, pCMV3-N) into HEK293 cells with α-Syn. Confocal microscopy carried out to demonstrate colocalization between α-Syn and SARS-CoV-2 proteins showed that α-Syn was colocalized with SARS-CoV-2 S protein and N protein around the nucleus (Figure 2A). To further investigate the endogenous interactions between α-Syn and SARS-CoV-2 proteins, we performed Co-IP in HEK293 cells co-transfected with α-Syn and SARS-CoV-2 S protein or N protein. Western blot analysis showed that anti-α-Syn antibody was able to immunoprecipitate SARS-CoV-2 N protein but not S protein, suggesting that α-Syn had a higher binding affinity to SARS-CoV-2 N protein in HEK293 cells (Figure 2B,C).

### 2.3. Elevated Expression of α-Syn by SARS-CoV-2 Proteins in HEK293 Cells

**Figure 3:**
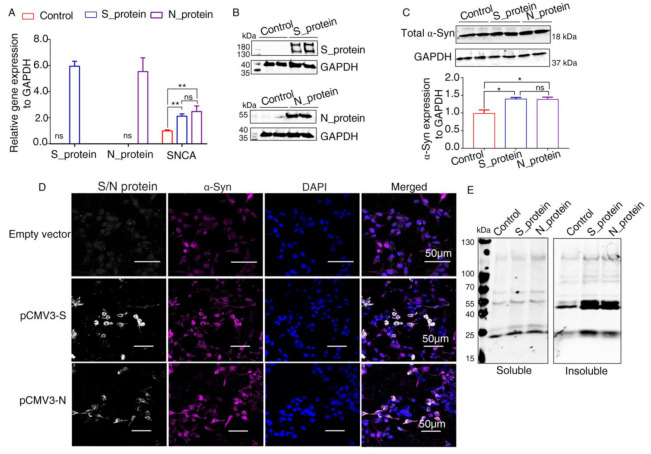
Elevated expression of α-Syn by SARS-CoV-2 proteins in HEK293 cells. (**A**) qRT-PCR analysis of the expression of α-Syn in HEK293 cells overexpressing S and N proteins. GAPDH was used as a loading control (*n* = 3). (**B**) Two days following transfection with pCMV3-S or pCMV3-N plasmids, the total protein from the HKE293 cells was extracted and detected by anti-SARS-CoV-2 S protein and anti-SARS-CoV-2 N protein. (**C**) Immunoblotting was performed using Syn1, an antibody that recognizes total α-Syn. GAPDH was used as a loading control. (**D**) The cells transfected with plasmids pCMV3-S or pCMV3-N for 48 h were fixed with paraformaldehyde containing 0.1% Triton X-100. Then, anti-α-Syn was detected by indirect immunofluorescence. DAPI was used to stain nuclei. (**E**) Western blotting detected “soluble” and “insoluble” fraction with an antibody against α-Syn (Syn1). *p* value represents results of One-way ANOVA. * *p* < 0.05; ** *p* < 0.01. “ns” means no significant difference.

It has been reported that α-Syn functions as a native antiviral factor in neurons, as shown by its increased neuronal expression following acute West Nile virus infection [22]. Given that SARS-CoV-2, similar to West Nile virus, is an enveloped, single-stranded, positive virus, we speculated that it would trigger elevated expression of α-Syn. We transfected plasmids expressing SARS-CoV-2 proteins into HEK293 cells for 48 h. qRT-PCR was performed to investigate *SNCA* expression at the transcriptional level. The results showed significantly increased *SNCA* expression in HEK293 cells overexpressing S and N proteins (Figure 3A). The Western blot results showed that S and N protein of SARS-CoV-2 were successfully expressed in HEK293 cells after transfection (Figure 3B). Further Western blot analysis confirmed that the total protein level of α-Syn (Syn1) was upregulated by SARS-CoV-2 proteins S and N (Figure 3C). However, there are no significant difference between S protein and N protein groups. These results were consistent with those of the immunofluorescence analysis by confocal microscopy (Figure 3D). The “soluble” and “insoluble” α-Syn species from the total protein were isolated by corresponding buffers in accordance with the protocol described above. As shown in Figure 3E, the accumulation of higher molecular weight α-Syn species of insoluble fraction was discovered in SARS-CoV-2 proteins-transfected cells. However, there were a few changes in the “soluble” fraction of α-Syn in SARS-CoV-2 proteins-overexpressing cells, suggesting that SARS-CoV-2 proteins accelerated the aggregation of α-Syn (Figure 3E).

### 2.4. SARS-CoV-2 Proteins Caused Lewy-Like Pathology in HEK293 Cells Overexpressing α-Syn

**Figure 4:**
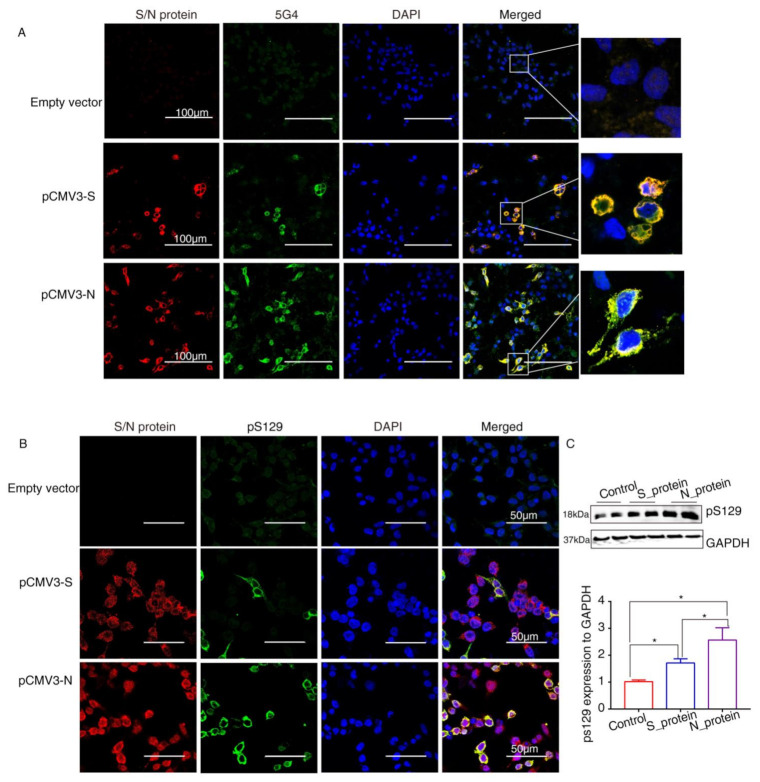
The SARS-CoV-2 proteins cause Lewy-like pathology in HEK293 cells overexpressing α-Syn. (**A**) Seventy-two hours after HEK293 cells overexpressing α-Syn had been transfected with pCMV3-S or pCMV3-N plasmids, they were fixed and stained with anti-α-Syn aggregate (5G4). Expression of aggregated α-Syn was observed using fluorescence confocal microscopy. (**B**) Seventy-two hours after HEK293 cells overexpressing α-Syn had been transfected with pCMV3-S or pCMV3-N plasmids, the cells were fixed and stained with anti-pS129-α-Syn. The results were observed under fluorescence confocal microscopy. (**C**) The total protein from the HKE293 cells was extracted and detected by anti-pS129-α-Syn. The expression levels were assessed by ImageJ. * *p* < 0.05.

α-Syn recruited into pathological inclusions undergoes extensive phosphorylation at Ser129 (pS129) [23]. The accumulation of phosphorylated α-Syn (*p*-α-Syn) reflects an intracellular modification. Previous studies have shown that approximately 90% of accumulated α-Syn in LBs of the brain is phosphorylated at p129, which is, therefore, considered pathology of PD [24]. To investigate pathological changes in SARS-CoV-2 proteins-overexpressing cells, specific antibodies against aggregated α-Syn (5G4) and pS129 were used to selectively recognize α-Syn pathology. Staining with the antibodies against SARS-CoV-2 proteins and 5G4 and pS129 α-Syn (Abcam, USA) in α-Syn-overexpressing HEK293 cells after transfection with SARS-CoV-2 proteins for 72 h showed that 5G4 and pSer129 α-Syn were increased by S protein and N protein and colocalized around the nucleus (Figure 4A,B). The Western blot results showed that the levels of pS129 α-Syn were significantly increased in SARS-CoV-2 proteins-transfected cells (Figure 4C), especially N_protein-transfected groups, indicating that SARS-CoV-2 proteins caused Lewy-like pathology in HEK293 cells when overexpressing α-Syn.

## 3. Discussion

PD or parkinsonism have been described associated with viral infections, such as influenza A, Epstein–Barr virus, varicella zoster, hepatitis C virus, Japanese encephalitis virus, West Nile virus, Coxsackie, and HIV [25,26,27]. There is growing epidemiological evidence that the pathological process of PD is accelerated in PD patients suffering from COVID-19 infection [28,29], suggesting a vicious cycle between PD and COVID-19. Neuroinvasion and neurotropism have been reported as common features of coronavirus infection [3]. Recent publications examining the localization of SARS-CoV-2 in individuals who died of COVID-19 demonstrated apparently low levels of SARS-CoV-2 RNA and proteins in the brain [30]. It has been confirmed that SARS-CoV-2 infection could be associated with various neurological distresses observed in the nervous system, such as headache, dizziness, impaired consciousness, acute cerebrovascular disease, epilepsy, and PNS-related manifestations such as hyposmia/anosmia, hypogeusia/ageusia, muscle pain, and Guillain-Barre syndrome [31]. Many of the neurological symptoms seen in COVID-19 patients, as well as the alterations in the gut microbiome, are also prevalent in patients with PD. Thus, we speculated that there are molecular interactions between COVID-19 and PD.

Many biological functions of proteins depend on the formation of protein–protein interactions. By performing protein–protein docking analysis, Danish et al. reported that the S1 RBD protein of SARS-CoV-2 could bind to a number of aggregation-prone heparin-binding proteins, including Aβ, α-Syn, tau, prion, and TDP-43 RRM. Especially, it has been verified that α-Syn has a more favorable binding affinity to SARS-CoV-2 S1 protein [13]. In test tube experiments between SARS-CoV-2 proteins and α-Syn, it has been shown that amyloid formation of α-Syn is accelerated by SARS-CoV-2 N protein, suggesting that SARS-CoV-2 might be connected to α-Syn [32]. In our study, we showed that α-Syn had a more favorable binding affinity to SARS-CoV-2 S protein and N protein. The direct interactions were further verified by confocal immunofluorescence and Co-IP in HEK293 cells, confirming the existing interaction between SARS-CoV-2 and α-Syn. However, we failed to pull down S protein by anti-α-Syn, likely due to a fragile connection that lacked salt bridge.

It has been suggested that SARS-CoV-2 infection invades the CNS by controlling the protein synthesis machinery, disturbs endoplasmic reticulum and mitochondrial function, and increases the accumulation of misfolded proteins, thereby activating protein aggregation, mitochondrial oxidative stress, and apoptosis, and leading to neurodegeneration [13]. In vitro and in vivo studies have confirmed that aggregation-prone protein, α-Syn, misfolding is a distinctive feature of PD. Overexpression of α-Syn in cells and animal models of PD has also resulted in cytotoxicity and recapitulation of several PD symptoms [33]. α-Syn is expressed in neurons both in the CNS and PNS as well as in erythrocytes and most immune cells [33]. Most recently, α-Syn has also been reported to function as a native antiviral factor within neurons, considering that its expression was increased and its aggregation was promoted after infection with West Nile virus [22], HINI [34], or H5NI [35]. In our study, we showed that the expression of α-Syn was upregulated in the used cell line. The results are consistent with the abovementioned studies [22,34,35]. Interestingly, elevated accumulation of α-Syn was detected in N protein- and S protein-overexpressing cells. α-Syn was prone to aggregation around the nucleus, and was colocalized with SARS-CoV-2 proteins. However, a study on seven COVID-19 patients with myoclonus, parkinsonism, and/or encephalopathy showed no differences in α-syn expression in serum and cerebrospinal fluid compared with healthy control subjects [36]. The limited amount of samples in that study might be the reason why their results do not support the hypothesis of α-Syn upregulation in humans with COVID-19 infection.

LBs are hallmark lesions in the brains of patients with PD, dementia with LB, and other neurodegenerative diseases. A large number of proteins have been identified in LBs, and the two most common ones are ubiquitin and α-Syn. In particular, phosphorylation at Ser129 is the dominant pathological modification of α-Syn in familial and sporadic LB diseases [23]. In the α-Syn-overexpressing cell line in our study, the aggregates and LBs-like pathology were observed after transfection with N protein and S protein. Elevated α-Syn expression may indeed serve as a protective factor against RNA viruses. However, it is unlikely that aggregated α-Syn contained within LBs is affective in restricting RNA viral replication. The mechanism by which SARS-CoV-2 induces α-Syn aggregates needs to be further studied.

PD has a complex and multifactorial etiology, and both the CNS and PNS are affected. Hence, a single pathogen is unlikely to be responsible for the entire pathogenesis of PD. There is also mounting evidence supporting the association of inflammation, mitochondrial dysfunction, autophagy deficiency, endoplasmic reticulum stress, and loss of proteostasis by SARS-CoV-2 infection with an elevated risk of PD later in life [4]. Several biochemical pathways, including oxidative stress, inflammation, and protein aggregation, show similarities between PD and COVID-19 [2].

We showed that SARS-CoV-2 protein could interact with α-Syn and induce LBs-like pathology in a cell line. Our finding that SARS-CoV-2 S and N proteins may induce endogenous α-Syn to form pathological aggregates support the epidemiological link between PD and COVID-19. Thus, our findings open up new avenues of research to understand mechanisms underlying the development PD on the basis of COVID-19.

## 4. Materials and Methods

### 4.1. Protein–Protein Docking

The RefSeq protein of *SNCA* (Accession: NP_000336.1), S protein (Accession: QOS45029), and N_protein (Accession: QOS44897.1) were obtained from the Protein database of NCBI (https://www.ncbi.nlm.nih.gov/protein/?term) (accessed on 21 February 2022). Structure of α-Syn bound to sodium dodecyl sulfate (SDS) micelles (PDB ID: 1XQ8) were used for protein and protein docking. This model is mainly based on the structural analog, full length human micelle-bound α-Syn. Its structure describes a-helices as the predominant secondary structure, besides random coil, which is highly similar to its native physiological conditions [37,38]. Protein–protein docking of SARS-CoV-2 S-RBD (PDB ID: 6M0J), N-CTD (PDB ID: 6WJI), and N-NTD (PDB ID: 6VYO) with α-Syn was performed on the HDOCK server (http://hdock.phys.hust.edu.cn/) (accessed on 21 February 2022), which is based on a hybrid algorithm of template-based modeling and ab initio free docking [39]. HDOCK finds homologous templates of the given sequences and then builds the structures from the monomer or complex templates for docking. The HDOCK server globally samples all possible binding modes between the two proteins through a fast Fourier transform (FFT)-based algorithm [40]. Then, all the sampled binding modes were evaluated by iterative knowledge-based scoring function ITScorePP [41]. Finally, the binding modes of macromolecules were evaluated by the binding energy and were ranked according to their docking energies. Then, the residual interactions of the three-dimensional model of protein complexes were analyzed through the PDBSUM server (http://www.ebi.ac.uk/pdbsum) (accessed on 21 February 2022), which is a web server that provides structural information including protein secondary structure, protein–ligand, and protein–DNA interactions [42]. The bonded and non-bonded interacting residues between the protein–protein interactions were examined. Furthermore, the structure model with the lowest docking energy score and the highest ligand root-mean-square deviation (RMSD) was selected to analyze the binding free energy scores (ΔG) and dissociation constant (Kd) using the PPA-Pred server (http://www.iitm.ac.in/bioinfo/PPA_Pred/) (accessed on 21 February 2022) [43].

### 4.2. Cell Culture and Transfection

Human kidney 293 (HEK293) cells purchased from ATCC (LGC Standards GmbH, Wesel, Germany) were used for cell experiments. The S protein and N protein cDNA cloned into pCMV3 (pCMV3-S, pCMV3-N) were purchased from SinoBiogical. The plasmid overexpressing *SNCA* (EF1α-SNCA) was constructed in our laboratory. The cells were cultured in Dulbecco’s modified Eagle medium (Gibco, Waltham, MA, USA) containing 10% fetal bovine serum (FBS; Sigma, St. Louis, MO, USA) at 37 °C in a humidified atmosphere containing 5% CO_2_. The plasmids were transfected using Lipofectamine 3000 (Thermo Fisher, Waltham, MA, USA) in accordance with the manufacturer’s protocol. Previously, the plasmids were mixed with Lipofectamine 3000 and added to the cells with fresh OPTI-MEM (Gibco, Waltham, MA, USA). After six hours, the culture was replaced with complete medium with 5% FBS. The cells were harvested after transfection with pCMV3-Sand pCMV3-N for further study.

### 4.3. RNA Extraction and Quantitative Real-Time PCR (qRT-PCR)

Total RNA was isolated from tissue samples and cultured cells using TRIzol (Sigma, USA) in line with the manufacturer’s instructions. Concentration and quality of the obtained RNA were determined using a NanoPhotometer (IMPLEN, München, Germany). The cDNA was generated from 2 μg total RNA using an iScript™ cDNA Synthesis Kit (Promega, Madison, WI, USA) in accordance with the manufacturer’s instructions. qRT-PCR was used to detect mRNA expression levels of target genes using an Eastep qPCR Master Mix (Promega, USA) and a CFX96 Real-Time PCR Detection System (Bio-Rad, Hercules, CA, USA). We used the following PCR cycling parameters: 10 min at 95 °C for initial denaturation, followed by 40 cycles of 30 s at 95 °C and 30 s at 60 °C. Sequences of the primers used in this experiment are listed in Supplementary Table S1. The comparative threshold (Ct) method was used to calculate the amount of cDNA normalized to the Ct of *GAPDH* gene. Relative gene expression levels were presented as relative quantification values calculated using the 2^−ΔΔCt^ method.

### 4.4. Western Blot Analysis

The cells were disrupted with ice-cold RIPA buffer with 2 mM PMSF and protease inhibitor cocktail (Merck, USA). The protein concentration was determined using a BCA Protein Assay Kit (Cwbio, Beijing, China). Total protein (20 μg) was separated by 10% TGX Stain-Free gels (Bio-Rad, USA) and transferred onto nitrocellulose membranes (Millipore, Burlington, MA, USA). Then, 5% skim milk was used to block the membranes for 1 h at room temperature. Subsequently, the membranes were incubated with primary antibodies at 4 °C overnight and then incubated with specific IRDye 800CW-conjugated antibodies (Odyssey, Lincoln, Nebraska, USA, 1:10,000) after washing with PBST three times. The information about the used antibodies is listed in Supplementary Table S2.

Soluble (Tx-soluble fraction) and insoluble (SDS-soluble fraction) α-Syn isolation was performed as previously described, with minor changes [44]. The lysates were centrifuged at 13,000× *g* for 30 min at 4 °C, and the supernatant was collected as a solution fraction. The pellet was washed twice with ice-cold PBS and resuspended in 2% SDS buffer (150 mM NaCl, 50 mM Tris pH 7.6, 2% SDS, 2 mM EDTA) supplemented with protease inhibitors (Merck, Kenilworth, NJ, USA) and phosphatase inhibitors (Merck, USA), were designated as “insoluble α-Syn.” after incubation on ice for 30 min. Soluble and insoluble α-Syn was subsequently immunoblotted as described above. The bands were visualized using the Odyssey imaging system (Licor, Lincoln, NE, USA). The densitometric analyses of the blots were performed using ImageJ software. GAPDH was used as a loading control. The one-way ANOVA was used to estimate the significance of difference in protein expression levels between groups. All tests were two sided and the level of statistical significance was set at *p* < 0.05, * *p* < 0.05, ** *p* < 0.01. Statistical analyses were performed using GraphPad Prism V 7.0.

### 4.5. Co-Immunoprecipitation Assay (Co-IP)

To further verify the interaction between α-Syn and N-protein and S-protein, Co-IP was performed as per the manufacturer’s manual (Pierce™ Classic Magnetic IP/Co-IP Kit, Boston, MA, USA). The HEK293 cells lysates co-transfected with α-Syn and N-protein or S-protein were lysed with IP lysis buffer (pH 7.4, 0.025 M Tris, 0.15 M NaCl, 0.001 M EDTA, NP40, 5% glycerol) and then incubated with antibody specific for α-Syn or with an IgG (used as negative control) in total of 10 mg overnight at 4 °C with shaking. The immune complex solution was incubated with protein A/G magnetic beads for 1 h at room temperature with stirring; the solution was then washed to remove the unbound immune complexes. The bound immune complexes were dissociated from the beads using a low-pH buffer and were analyzed using Western blotting.

### 4.6. Confocal Immunofluorescence Assays

The plasmids expressing α-Syn and SARS-CoV-2 proteins were transfected to HEK293 cells by Lipofectamine 3000. Immunofluorescence was performed in accordance with a previously established protocol [45]. The cells plated in 24 wells were fixed with 4% paraformaldehyde and 4% sucrose at room temperature for 30 min, followed by permeabilization in 0.1% Triton X-100. After fixation and permeabilization, the cells were incubated with primary antibody at 4 °C overnight. After washing with PBS, the cells were incubated with Alexa Fluor 488-conjugated goat anti-mouse and Alexa Fluor 594-conjugated goat anti-rabbit secondary antibodies. The samples were observed under a laser scanning confocal microscope (Leica TCS SP8, Wetzlar, Germany). The information related to the used antibodies is listed in Supplementary Table S2.

## 5. Conclusions

We concluded that SARS-CoV-2 proteins interacted with α-Syn, and elevated its expression and LBs-like pathology in HEK293. The molecular interaction between SARS-CoV-2 proteins and α-Syn offered new insights into the mechanism underlying the development of PD on the background of COVID-19.

## Figures and Tables

**Table 1:** Molecular docking of SARS-CoV-2 proteins to α-Syn determined by HDOCK server.

Protein–Protein Complex	Docking Score	ΔG (kcal mol^−1^)	Kd (M)
N-CTD-α-Syn	−252.56	−8.63	4.72 × 10^−07^ M
N-NTD-α-Syn	−209.03	−9.09	2.17 × 10^−07^ M
S1_RBD-α-Syn	−243.43	−10.68	1.46 ×10^−08^ M
S1_RBD-ACE2	−291.07	−12.95	3.17 × 10^−10^ M

## Data Availability

Data is contained within the article.

## Supplementary Materials

The following supporting information can be downloaded at: https://www.mdpi.com/article/10.3390/ijms23063394/s1.

## Abbreviations

PD	Parkinson’s disease
α-Syn	Alpha-synuclein
SARS-CoV-2	severe acute respiratory syndrome coronavirus 2
COVID-19	2019 novel coronavirus disease
LBs	Lewy bodies
N	nucleocapsid
S	spike
RBD	receptor-binding domain
ACE2	angiotensin-converting enzyme 2
NTD	N-terminal domain
CTD	C-terminal domain
PNS	peripheral nervous systems
CNS	central nervous systems
△G	binding free energy scores
Kd	dissociation constant
qRT-PCR	Quantitative real-time PCR
pS129	phosphorylation at Ser129
HKE29	human kidney 293
