# Supplementary material for: SARS-CoV-2 Proteins Interact with Alpha Synuclein and Induce Lewy Body-like Pathology In Vitro
Source: Int J Mol Sci. 2022 Mar 21;23(6):3394. doi: 10.3390/ijms23063394 (PMC8949667; doi:10.3390/ijms23063394)
Supplement: Supplementary file 1 [file ijms-23-03394-s001.zip › ijms-1631277-supplementary.pdf]

Supplementary Materials

Table S1. Related primer sequences to qRT-PCR.

Gene Name	Primers
SNCA	Forward 5′- GTGGCTGCTGCTGAGAAAAC
	Reverse 5′- CACCACTGCTCCTCCAACAT
GAPDH	Forward 5′- TGCACCACCAACTGCTTAGC
	Reverse 5′- GGCATGGACTGTGGTCATGAG
S protein	Forward 5′- CCTGGTTCCATGCCATCCAT
	Reverse 5′- TCCAGGGTGGTGCCAAAAAT
N protein	Forward 5′- ACCAAAGGACCACATTGGCA
	Reverse 5′- ATAGAAGCCCTTTGGCAGGG

Table S2. The antibodies associated with this research.

Primary Antibodies	Type	Source
anti- a-Syn	mouse monoclonal (LB509)	Abcam
anti- a-Syn	rabbit monoclonal (ab13850)	Abcam
anti-S_protein	rabbit polyclonal(40591-T62)	SinoBiological
anti-S_protein	mouse monoclonal(40591-MM42)	SinoBiological
anti- N_protein	rabbit monoclonal(40143-R019)	SinoBiological
anti- N_protein	mouse monoclonal(40588-MM137)	SinoBiological
anti – p129-αSyn	rabbit monoclonal(ab124821)	Abcam
anti-GAPDH	rabbit polyclonal(10494-1-AP)	proteintech
anti-aggregated a-Syn (5G4)	mouse monoclonal l((MABN389))	Millipore
iFluorTM 633	goat anti-rabbit	Abcam
Alexa Fluor 552	goat anti-mouse	Abcam
Alexa Fluor 552	goat anti-rabbit	Abcam
IRDye-conjugated secondary antibodies	goat anti-mouse	Odyssey
IRDye-conjugated secondary antibodies	goat anti-rabbit	Odyssey
